# The missing slope: paradoxical shortening of activated partial thromboplastin time in a patient on unfractionated heparin therapy

**DOI:** 10.11613/BM.2021.021003

**Published:** 2021-06-15

**Authors:** Ivana Lapić, Ana Lončar Vrančić, Désirée Coen Herak, Dunja Rogić

**Affiliations:** Department of Laboratory Diagnostics, University Hospital Centre Zagreb, Zagreb, Croatia

**Keywords:** pre-analytical phase, hemostasis, activated partial thromboplastin time, unfractionated heparin

## Abstract

This case report describes false shortening of activated partial thromboplastin time (aPTT) due to erroneous optical reading of the clotting point in the presence of unfractionated heparin (UFH), and a biphasic waveform. Activated partial thromboplastin time performed on a coagulometer with photo-optical detection yielded an ambiguous clotting curve characterized by an early and steady decrease in light transmittance throughout the whole measuring range, with the clotting point read at 65 seconds. Further investigations included measurement of aPTT by means of a mechanical clot detection method as well as determination of another heparin-sensitive coagulation assay, that is thrombin time (TT), both being unmeasurably prolonged (> 150 seconds). Communication with clinicians revealed that the patient was on continuous UFH therapy and had an underlying sepsis, with highly elevated C-reactive protein (289 mg/L). The aPTT measurements requested at three timepoints later during the same day revealed gradual aPTT shortening and unveiled a peculiar biphasic waveform pattern. In this case, unmeasurably prolonged aPTT due to UFH therapy was masked by a biphasic aPTT curve pattern making only the first slope of the biphasic waveform visible within the measuring range. The early decrease in plasma light transmittance mimicked optical changes related to clot formation, thus causing erroneous optical reading and yielding a falsely shortened aPTT. This case emphasizes that such a pattern should be carefully inspected, especially when a combination of a critically ill condition and UFH therapy is present, in order to prevent erroneous reporting of aPTT and potential adverse effects on patient care.

## Introduction

Activated partial thromboplastin time (aPTT) is a widely used coagulation test intended for screening of clotting factor deficiencies within intrinsic and common coagulation pathways, for detecting presence of circulating inhibitors such as lupus anticoagulant, and for therapeutic monitoring of unfractionated heparin (UFH). The principle of the test is based on measuring platelet-poor plasma clotting time after addition of contact activators, phospholipids and calcium. The test is nowadays usually performed on fully automated or semiautomated coagulometers with photo-optical or mechanical detection. Clot formation is identified by a change in optical density (OD) of the reaction mixture and aPTT is defined as elapsed time in seconds when the reaction mixture OD has exceeded a predefined threshold (*1*). Interfering substances that change the sample OD inevitably influence optical coagulometer readings (*2*). Besides the well-documented effects of hemolysis, icterus and lipemia on aPTT determination, it has been reported that in some critically ill conditions deviations from the normal sigmoidal OD curve pattern can occur, leading to erroneous optical readings (*2*, *3*). The most common example is the biphasic waveform which can be encountered in patients with hemostatic dysfunction caused by disseminated intravascular coagulation (DIC) and sepsis (*1*, *4*).

Hereby, we present an unusual case of a falsely shortened aPTT result due to erroneous optical reading of the clotting point in the presence of both UFH and a biphasic waveform.

## Case report

Activated partial thromboplastin time was requested as part of routine laboratory monitoring of UFH therapy. A blood sample drawn into a 4.5 mL 0.105 M (3.2%) trisodium citrate vacutainer (Becton Dickinson, Plymouth, United Kingdom) was promptly delivered to the Department of Laboratory Diagnostics, University Hospital Centre Zagreb, Croatia. Upon receipt, the sample was processed without delay according to standard laboratory operation procedure, *i.e.* visual inspection for clots followed by centrifugation of the sample tube at 2000xg for 15 minutes at room temperature within one hour from blood collection. The obtained plasma was clear, without visible turbidity or haemolysis.

**Figure 1:**
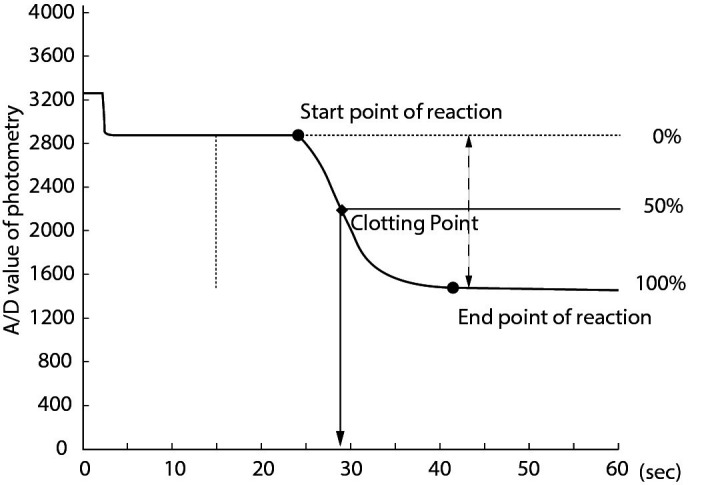
A normal, sigmoidal activated partial thromboplastin time (aPTT) clotting curve with an initial plateu, followed by decrease in light transmittance due to clot formation and a final plateau once the clot formation is completed. The clotting point is set at 50% of the descending clotting curve.

Activated partial thromboplastin time determination was performed by coagulometric method using Dade Actin FS and Calcium Chloride (Siemens Healthcare, Marburg, Germany) with photo-optical clot-detection system on the Sysmex CS-5100 automated coagulation analyser (Siemens Healthcare, Marburg, Germany) according to manufacturer’s recommendations. A normal aPTT measurement curve is shown in Figure 1.

**Figure 2:**
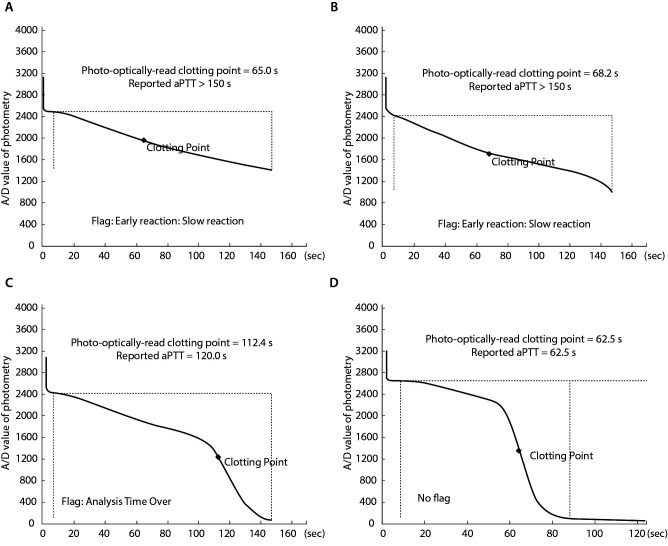
aPTT clotting curves with clotting points obtained on the coagulometer with photo-optical detection and accompanying analyser flags at (A) initial blood draw, after (B) 3 hours, (C) 7 hours and (D) 11 hours; The biphasic waveforms are in (A) and (B), and to a less extent in (C), masked by a prolonged aPTT due to unfractionated heparin, and caused erroneous optical reading of the clotting point; Reported aPTT values were in cases (A) and (B) obtained using the mechanical clot detection method, while for (C) by manual reading of the clotting point at 50% of the secondary decreasing slope of the clotting curve obtained photo-optically; (D) represents a typical biphasic aPTT waveform with proper photo-optical reading of the clotting point which corresponds to the reported aPTT. aPTT - activated partial thromboplastin time.

An ambiguous aPTT result was obtained and therefore numerical result was omitted and replaced by asterisks (******). The analyser software generated a flag indicating that further review by laboratory staff was required. The clotting point read by the analyser was at 65 seconds (reference interval: 24-33 seconds). Detailed visual inspection revealed an atypical clotting curve as shown in Figure 2A, accompanied by a flag indicating *Early reaction error: Slow reaction,* which raised a suspicion about a non-specific initial decrease in light transmittance and interference in optical reading.

Therefore, we additionally performed aPTT measurement by a mechanical clot detection method utilizing the semiautomated dual-channel BEHRING Fibrintimer (Sysmed Lab, Europe Inc., Poland) analyser and using the same reagents as for the optical method. No clot formation was detected and an unmeasurable aPTT was obtained (> 150 seconds).

Since the most common cause of aPTT prolongation is heparin presence, we performed another heparin-sensitive coagulation assay - thrombin time (TT). The measurement was done utilizing Test Thrombin reagent (Siemens Healthcare, Marburg, Germany) on the Sysmex CS-5100 analyser. An unmeasurably prolonged result was observed (> 150 seconds, reference interval: 16-18 seconds).

Further investigation of the case, done through communication with clinicians, revealed that the patient was treated with extracorporeal membrane oxygenation (ECMO) after a cardiorespiratory arrest and therefore inevitably required continuous UFH infusion. The patient’s condition was additionally complicated by sepsis, as confirmed by blood culture, and by a markedly elevated C-reactive protein (CRP) of 289 mg/L (reference interval: < 5 mg/L). Procalcitonin was not requested since it was not needed for case clarification.

Furthermore, aPTT for the same patient was requested three more times during the same day, *i.e.* 3, 7 and 11 hours after the initial blood draw, with clotting curves as shown in Figures 2B-2D, respectively. These subsequent analyses revealed gradual aPTT shortening and unveiled a peculiar biphasic waveform pattern which was not visible in the initial aPTT determination due to unmeasurably prolonged aPTT. Figure 2D represents a typical biphasic aPTT waveform.

## What happened?

Unmeasurably prolonged aPTT due to UFH therapy was masked by a biphasic aPTT curve pattern, making only the first slope of the biphasic waveform visible within the measuring range. Early decrease in plasma light transmittance mimicked optical changes related to clot formation, thus causing erroneous optical reading and yielding a falsely shortened aPTT.

## Discussion

Automated coagulation analysers with photo-optical detection measure a change in light transmittance in the reaction mixture during the entire clotting process and yield an appropriate curve. Regular clotting curves have an initial, horizontal plateau with no change in light transmittance, followed by an abrupt decrease once the coagulation process begins due to fibrin polymerisation, ending with a final, steady-state plateau when clot formation is completed, eventually forming a sigmoid-shaped curve (*1*, *4*). Abnormal biphasic clotting curves due to non-clot formation-related changes of light transmittance, as those described herein, trigger a particular analyzer flag and inevitably require visual inspection by the operator. Typical biphasic aPTT waveforms are characterized by absence of the initial plateau coupled with immediate, steady decline in light transmittance in the early precoagulation phase, followed by a secondary, sigmoidal decreasing slope when clot formation actually occurs (*5*). This is thought to be attributable to the rapid formation of Ca^2+^-dependent macroscopic complex consisting of CRP and very low density lipoprotein (VLDL) that is triggered by recalcification of citrate plasma through addition of aPTT reagents. This immediately causes changes in the reaction mixture OD, reflected as an early drop in light transmittance (*5*). Although the underlying mechanism has not been completely elucidated, it was suggested that both a substantially elevated CRP and adequate levels of VLDL that exhibit a high surface activity for prothrombin activation are necessary for the complex formation (*1*, *5*). The reaction itself requires calcium ions, but does not depend on the composition of the aPTT reagent. Such pattern can be seen in critically ill patients with conditions that often lead to DIC, the most common being sepsis, but also in cases of trauma, severe burns or malignancies (*4*). Earlier studies have shown that the presence of a biphasic waveform is associated with increased mortality rates and can be an early indicator of adverse outcomes (*4*, *6*-*8*). Given the diagnosis of sepsis and a concomitant high CRP level in the studied case, it can be highly suspected that the observed biphasic waveform resulted from the formation of CRP-VLDL complexes once the plasma recalcification had occurred. Unlike other described cases, this one was additionally complicated by UFH therapy that caused aPTT prolongation, masked the biphasic shape of the clotting curve and paradoxically yielded false shortening of aPTT when measured photo-optically.

The nowadays less frequently used semiautomated mechanical clot detection method was a valuable alternative in our case since it simply measures the clotting endpoint by detecting changes in the movement of a steel ball or rod in the reaction cuvette, and is therefore not susceptible to optical interferences (*3*). This method revealed an unmeasurable aPTT, a result which was consistent with UFH therapy that the patient was receiving during ECMO support. The presence of UFH in the sample was further supported by unmeasurably prolonged TT. Heparin binds to antithrombin and in that way exhibits its inhibitory effect on thrombin, prolonging both aPTT and TT (*9*). Neither D-dimers nor fibrinogen were concomitantly determined to evaluate the possible development of DIC, which could have elucidated the underlying pathophysiological cause of the interference.

## What YOU should/can do in your laboratory to prevent such errors

A biphasic aPTT clotting curve should be considered when an early and progressive decrease in light transmittance is present.
Such pattern should serve as an alert for a possible underlying interference in optical reading that might require manual reading of the clotting point or even adoption of a mechanical clot detection method.
Special attention should be given to the combination of a critically ill condition, most commonly sepsis or DIC, and UFH therapy which can result in an ambiguous biphasic aPTT waveform where the slope related to clot formation is not present due to the anticoagulant effect of UFH.
Biological interference should not be overlooked as a potential source of preanalytical errors in coagulation testing.
